# Retained fragmented Raytec gauze eluding an alleged correct postoperative count following cardiothoracic surgery: case report of a rare “never event”

**DOI:** 10.1186/s13037-025-00472-w

**Published:** 2025-12-20

**Authors:** Marco Lizwan, Philip Y. K. Pang

**Affiliations:** Department of Cardiothoracic Surgery, National Heart Centre Singapore, 5 Hospital Dr, Singapore, 169609 Singapore

**Keywords:** Gossypiboma, Radio-opaque marker, Raytec gauze, Retained surgical items, Surgical safety

## Abstract

**Introduction:**

Retained surgical items are rare yet serious complications that may occur despite correct instrument and sponge counts. Surgical sponges remain the most frequently retained items, with gauze marker strand retention being an uncommon mechanism.

**Case presentation:**

A 69-year-old male with severe mitral regurgitation and triple-vessel coronary artery disease underwent mitral valve replacement with coronary artery bypass grafting. All surgical counts were verified as correct at the conclusion of surgery. Postoperative chest radiography, however, revealed two linear radio-opaque foreign bodies near the left lower sternum. Mediastinal re-exploration identified two radio-opaque strands, measuring 3.0 cm and 2.0 cm, attached to the left lower chest wall near the internal mammary artery stump. These were confirmed to be detached marker strands from Raytec gauzes. The patient’s recovery was uneventful and he was discharged well.

**Conclusion:**

This case illustrates that retained gauze fragments can occur despite correct counts due to fragmentation of radiopaque markers. Surgeons and operating room nurses should inspect gauzes for integrity, maintain vigilance when manipulating sponges in confined operative fields, and consider adjunct technologies such as radiofrequency or barcode tracking. In high-risk surgeries, postoperative imaging may be warranted even with accurate counts to ensure patient safety.

## Introduction

Retained surgical items are among the most avoidable yet serious complications in operative practice [1]. Classified as sentinel or “never events”, retained surgical items are associated with patient morbidity, medicolegal implications and reputational consequences for surgical teams [2, 3]. Although the implementation of standardized counting systems and the use of radiopaque markers have markedly reduced the incidence of retained surgical items, they remain a persistent issue [4]. A significant proportion of retained surgical items are reported even when surgical counts are documented as correct [1]. The majority involve retained surgical sponges or gauze, collectively termed gossypiboma, which can manifest as infection, abscess or chronic inflammation if not identified early [5]. Although classical retained surgical items typically refer to the whole surgical sponges, an increasing number of reports have highlighted retention of smaller components [6, 7].

This report describes a rare case of retained Raytec gauze marker strands discovered after combined mitral valve replacement and coronary artery bypass grafting, despite correct intraoperative counts. The case underscores that fragmentation of gauze marker lines can result in retention of small but radiographically visible foreign bodies. We discuss the mechanisms underlying such events and highlight preventive strategies to strengthen existing patient safety systems.

## Case presentation

**Fig. 1:**
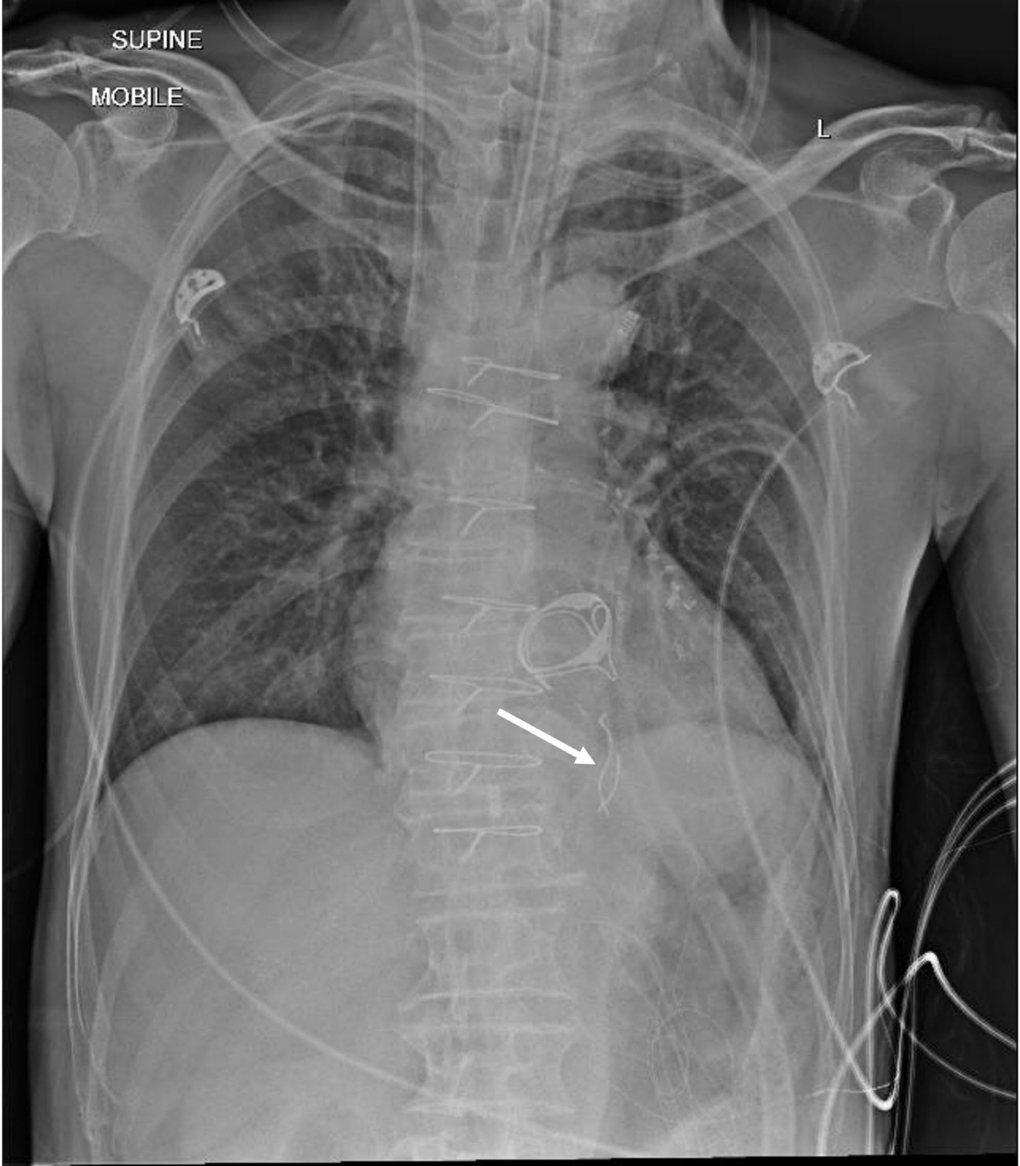
Routine postoperative chest radiograph demonstrating two curvilinear radio-opaque densities projected over the left cardiac silhouette (arrow). These were not visible externally and raised suspicion for retained intra-thoracic foreign bodies

A 69-year-old male with severe mitral regurgitation and triple-vessel coronary artery diseaseunderwent elective mitral valve replacement and concomitant coronary artery bypass grafting. All surgical counts were verified as correct at the conclusion of the surgery. On arrival at the intensive care unit, a routine postoperative chest radiograph demonstrated two linear radio-opaque densities at the left lower sternal region (Fig. 1). Although the counts were correct, the radiographic findings prompted concern for retained surgical material. The patient was returned to the operating theatre for mediastinal re-exploration.

**Fig. 2:**
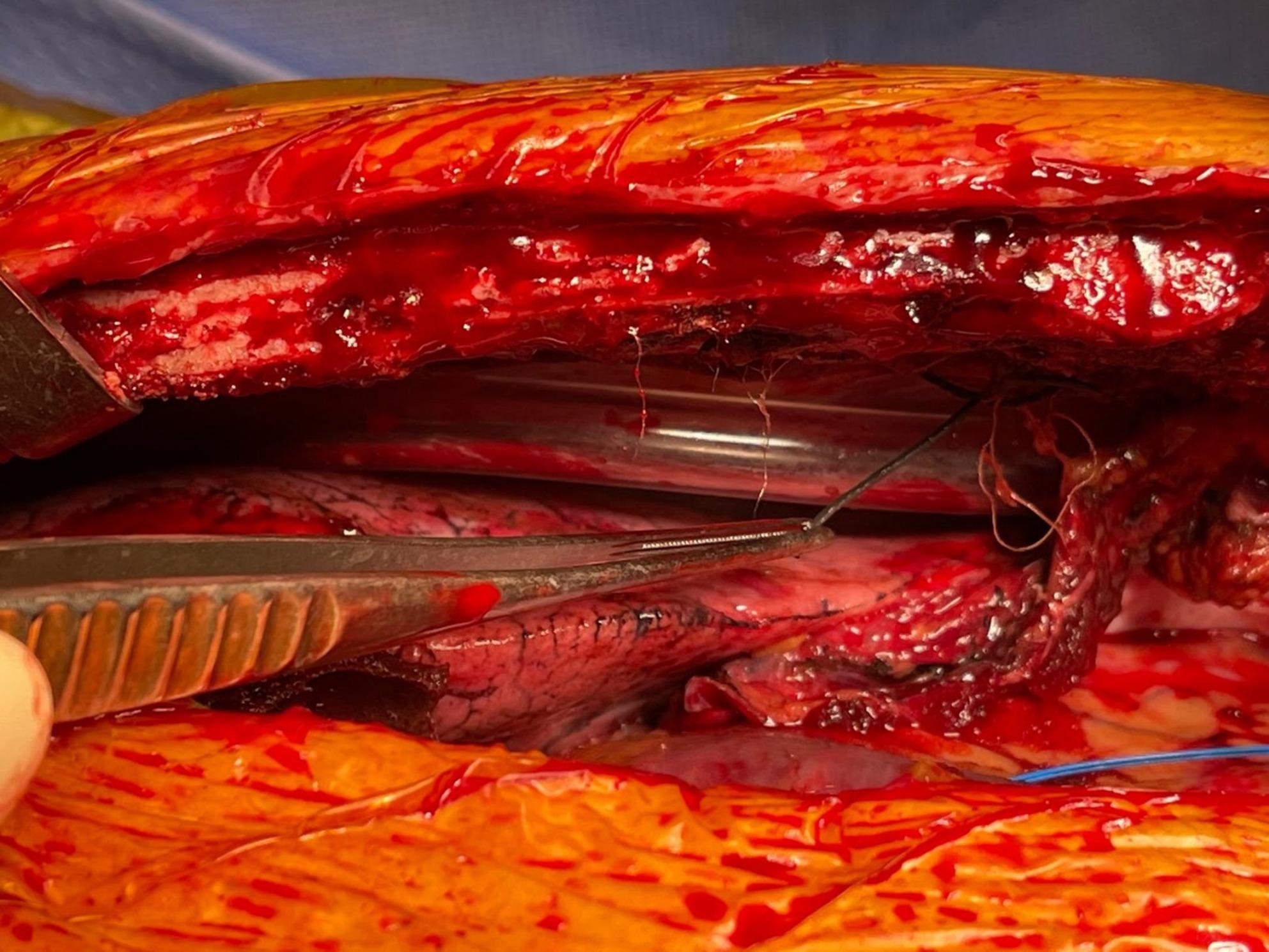
Intraoperative photograph showing detached radio-opaque marker strands adherent to the left lower chest wall adjacent to the left internal mammary artery stump, partially obscured by the chest drain, corresponding to the radiographic findings

Intraoperatively, two linear radio-opaque strands measuring 3.0 cm and 2.0 cm were identified attached to the left lower chest wall adjacent to the left internal mammary artery stump (Fig. 2). These matched the dimensions and location of the radiographic findings. The fragments were confirmed to be radio-opaque marker strands detached from Raytec gauzes. The surrounding tissues were clean, and there was no evidence of infection or inflammation. The fragments were removed completely prior to routine sternal closure.

His postoperative course was uneventful except for transient atrial fibrillation, which resolved with medical management. The patient was discharged home on postoperative day 8. At scheduled follow-up visits at 2 weeks and 3 months after surgery, he remained asymptomatic, clinically well, and without radiographic or wound-related complications.

## Discussion

Retained surgical items continue to represent a significant patient safety concern despite standardized counting protocols and the adoption of safety checklists. Surgical sponges remain the most frequently retained items reported across institutions, with the majority of cases involving whole sponges rather than isolated components such as radiopaque marker strands. Traditional risk factors include emergency procedures, unplanned intraoperative changes, prolonged operative duration, and patient obesity. However, retained surgical items may still occur even when counts are correct, as described in landmark studies by Gawande et al. and others, demonstrating the inherent limitations of counting alone [1, 8, 9]. Our case illustrates a rare but important mechanism – fragmentation of radiopaque gauze markers – that can escape detection through conventional safety systems.

While adjunct technologies such as barcoded sponges and radiofrequency identification (RFID) systems have been shown to significantly reduce retained surgical item rates, they depend on tagging devices of a minimum physical size to ensure reliable detection [9–11]. Because radiopaque marker filaments in Raytec gauze are extremely thin, they are not amenable to RFID or barcode-based tracking, leaving a detection gap for small fragments. This limitation has been acknowledged in prior retained surgical item prevention literature, which emphasizes that technological solutions cannot fully replace surgical vigilance [2, 12, 13].

Material redesign offers a promising avenue to address this gap. Manufacturers have begun modifying the tensile strength and bonding of radiopaque polypropylene filaments to reduce the risk of detachment during vigorous manipulation. Historical analyses of retained sponge cases have identified marker–gauze separation as a plausible but previously under-recognized mechanism of retained surgical item [13, 14]. More recent engineering approaches include embedding radiopaque material within a woven mesh rather than using a separate filament, thereby reducing shear-related failure. These developments remain in evolution, but parallels can be drawn from broader retained surgical item prevention efforts that demonstrate how engineering innovation can meaningfully improve reliability [12, 15].

Another strategy involves selective intraoperative imaging. Although routine radiography for all procedures is impractical, targeted low-dose imaging during high-risk operations – such as cardiac surgery or extensive mediastinal dissection – may identify retained small radiopaque fragments that escape detection by manual counts. Studies evaluating improved digital radiography and AI-assisted interpretation suggest improved sensitivity for thin foreign bodies, offering future promise for early detection [13, 15]. Furthermore, human-factor interventions such as meticulous visual inspection of sponge integrity before discard, cross-verification among operating room staff, and heightened awareness of marker fragmentation can serve as critical safeguards in preventing similar events [2, 12, 16].

Our case underscores that radiopaque marker strand detachment, although rare, is a genuine retained surgical item mechanism that warrants broader recognition. A multimodal prevention strategy – combining improved material design, selective intraoperative imaging, and strengthened human-factor practices – may provide the most comprehensive protection against such occurrences. Collaboration between clinicians, engineers, and industry partners will be essential for addressing this subtle but clinically significant patient safety risk.

## Conclusion

This case illustrates a rare instance of retained Raytec gauze marker strands following mitral valve replacement and coronary artery bypass grafting, discovered despite correct surgical counts. Fragmentation of radiopaque markers can lead to the retention of small but clinically significant foreign bodies. Surgical teams should incorporate integrity checks for all sponges, consider radiographic verification in high-risk operations, and adopt adjunctive technologies to enhance safety. Continuous vigilance and adherence to multimodal safety strategies remain crucial in eliminating retained surgical items.

## Data Availability

Additional data are available from the corresponding author on reasonable request.

## Abbreviations

RFID: Radiofrequency identification
